# Targeting PP2A activates AMPK signaling to inhibit colorectal cancer cells

**DOI:** 10.18632/oncotarget.21336

**Published:** 2017-09-28

**Authors:** Cuiping Dai, Xuning Zhang, Da Xie, Peipei Tang, Chunmei Li, Yi Zuo, Baofei Jiang, Caiping Xue

**Affiliations:** ^1^ Faculty of Health, Jiangsu Food and Pharmaceutical Science College, Huaian, China; ^2^ Huaian Key Laboratory Of Gastrointestinal Cancer, Jiangsu College of Nursing, Huaian, China; ^3^ Oncology Department, The Second Affiliated Hospital of Nanjing Medical University, Nanjing, China; ^4^ Department of Medicine, Xinglin College, Nantong University, Nantong, China; ^5^ Gastrointestinal Surgery, The First People's Hospital of Huaian City, Huaian, China

**Keywords:** protein phosphatase 2A (PP2A), LB-100, colorectal cancer (CRC), AMP-activated protein kinase (AMPK) and mTOR

## Abstract

LB-100 is a novel PP2A inhibitor. Its activity in human colorectal cancer (CRC) cells was tested. The *in vitro* studies demonstrated that LB-100 inhibited survival and proliferation of both established CRC cells (HCT-116 and HT-29 lines) and primary human colon cancer cells. Further, LB-100 activated apoptosis and induced G1-S cell cycle arrest in CRC cells. LB-100 inhibited PP2A activity and activated AMPK signaling in CRC cells. AMPKα1 dominant negative mutation, shRNA-mediated knockdown or complete knockout (by CRISPR/Cas9 method) largely attenuated LB-100-induced AMPK activation and HCT-116 cytotoxicity. Notably, microRNA-17-92-mediated silence of PP2A (regulatory B subunit) also activated AMPK and induced HCT-116 cell death. Such effects were again largely attenuated by AMPKα mutation, silence or complete knockout. *In vivo* studies showed that intraperitoneal injection of LB-100 inhibited HCT-116 xenograft growth in nude mice. Its anti-tumor activity was largely compromised against HCT-116 tumors-derived from AMPKα1-knockout cells. We conclude that targeting PP2A by LB-100 and microRNA-17-92 activates AMPK signaling to inhibit CRC cells.

## INTRODUCTION

Recently, colorectal cancer (CRC) studies have been focusing on molecule-targeted therapy [1, 2] and exploring novel chemo-preventive agents [3–6]. Protein phosphatase 2A (PP2A) is a well-established serine/threonine phosphatase [7–9], which is extremely important in regulation of mitotic progression and DNA damage responses [10]. Although PP2A was traditionally viewed as a tumor suppressor [10, 11], recent cancer studies have indicated that PP2A inhibition could inhibit cancer cells via driving senescent cancer cells into mitosis or promoting cancer cell death and apoptosis [12, 13]. Several known PP2A inhibitors were shown to inhibit cancer cell proliferation and/or to induce cancer cell apoptosis [12, 13]. LB-100 is a novel small-molecule PP2A inhibitor [12–14]. Its potential activity in human CRC cells is tested here.

One important PP2A substrate protein kinase is AMP-activated protein kinase (AMPK) [15–17], which is composed of the catalytic α and regulatory β and γ subunits. AMPK is the master energy sensor, whose activation maintains energy homeostasis under stress conditions [18–20]. Recent studies have proposed a tumor-suppressing function of activated AMPK [4, 21–24]. AMPK inhibits its substrates acetyl-CoA carboxylase (ACC) [19, 25] and mammalian target of rapamycin (mTOR) complex 1 (mTORC1) to block fatty acid and protein synthesis [26]. Activated AMPK could also induce p53 stabilization and activation, thus causing cell arrest and apoptosis [27]. Further, AMPK activation could trigger autophagic cell death via multiple mechanisms involving mTOR, ULK1, and VPS34 [28, 29]. Activation of AMPK by a number of anti-cancer agents and natural occurring compounds efficiently inhibits human CRC cells [4, 21, 22]. In the current study, we show that targeting PP2A by LB-100 and microRNA-17-92 (“miR-17-92”) activates AMPK signaling, which in turn inhibits CRC cells *in vitro* and *in vivo*.

## RESULTS

### LB-100 inhibits survival and proliferation of CRC cells

**Figure 1:**
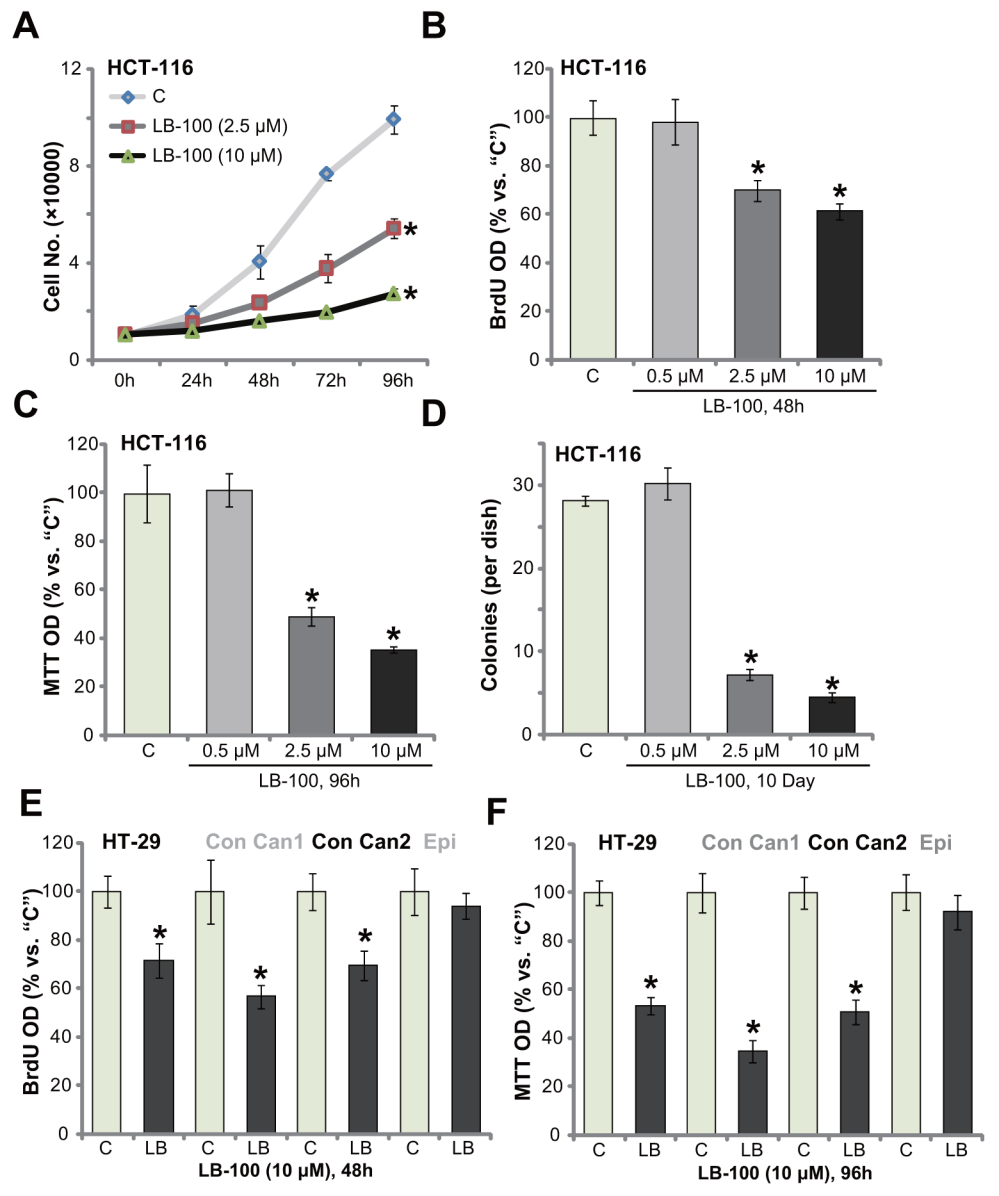
LB-100 inhibits survival and proliferation of CRC cells The established human CRC cells (HCT-116 and HT-29 lines), the primary-cultured human colon cancer cells (two lines, “Con Can1/2”), or the primary-cultured colon epithelial cells (“Epi”) were treated with LB-100 at designated concentration, cells were further cultured for applied time; Cell proliferation **(A, B** and **E)** and cell survival **(C, D** and **F)** were examined by the assays mentioned in the text. Error bars indicate standard deviation (SD). “C” stands for untreated control group. ^*^p<0.05 vs. “C” group. Experiments in this figure were repeated five times, and similar results were obtained.

In order to test the potential activity of LB-100 on human CRC cells, the established HCT-116 HCC cells [5] were cultured in complete medium, and were treated with LB-100 at different concentrations. Simple cell counting assay results in Figure 1A displayed that treatment with LB-100, at 2.5 μM and 10 μM, significantly inhibited HCT-116 cell proliferation. The number of HCT-116 cells was decreased following LB-100 treatment (Figure 1A). LB-100 at 10 μM was more potent than 2.5 μM in inhibiting HCT-116 cell proliferation, displaying a concentration-dependent response (Figure 1A). BrdU incorporation is a well-utilized marker of cell proliferation. Results in Figure 1B confirmed that LB-100 treatment (2.5 μM and 10 μM) significantly decreased BrdU ELISA (enzyme-linked immunosorbent assay) optic density (OD) in HCT-116 cells, confirming its anti-proliferative activity. To test cell viability, the routine MTT tetrazolium assay was performed. As demonstrated, LB-100 treatment (2.5 μM and 10 μM, 96 hours) largely inhibited survival (“MTT OD”) of HCT-116 cells (Figure 1C). Additionally, the number of viable HCT-116 cell colonies was also significantly decreased following LB-100 treatment (2.5 μM and 10 μM, renewed every two days) (Figure 1D). Notably, the anti-survival activity by LB-100 was also concentration-dependent. At a relative low concentration (0.5 μM), LB-100 failed to decrease HCT-116 cell survival (Figure 1C and 1D).

We also examined the potential effect of this novel PP2A inhibitor [13, 30, 31] in other CRC cells: including the established HT-29 cells and two lines of primary human colon cancer cells (“Con Can1/2”). BrdU ELISA proliferation assay results (Figure 1E) and MTT viability assay results (Figure 1F) displayed that treatment with LB-100 (10 μM) dramatically inhibited survival and proliferation of the tested CRC cells, as the BrdU ELISA OD and MTT OD were both decreased following the LB-100 treatment (Figure 1E and 1F). Intriguingly, the very same LB-100 (10 μM) treatment failed to inhibit proliferation and survival of the primary human colon epithelial cells (“Epi”) (Figure 1E and 1F). BrdU ELISA OD and MTT OD were not significantly decreased after LB-100 treatment (Figure 1E and 1F). Collectively, the results indicate that LB-100 inhibits survival and proliferation of human CRC cells.

### LB-100 activates apoptosis in CRC cells

**Figure 2:**
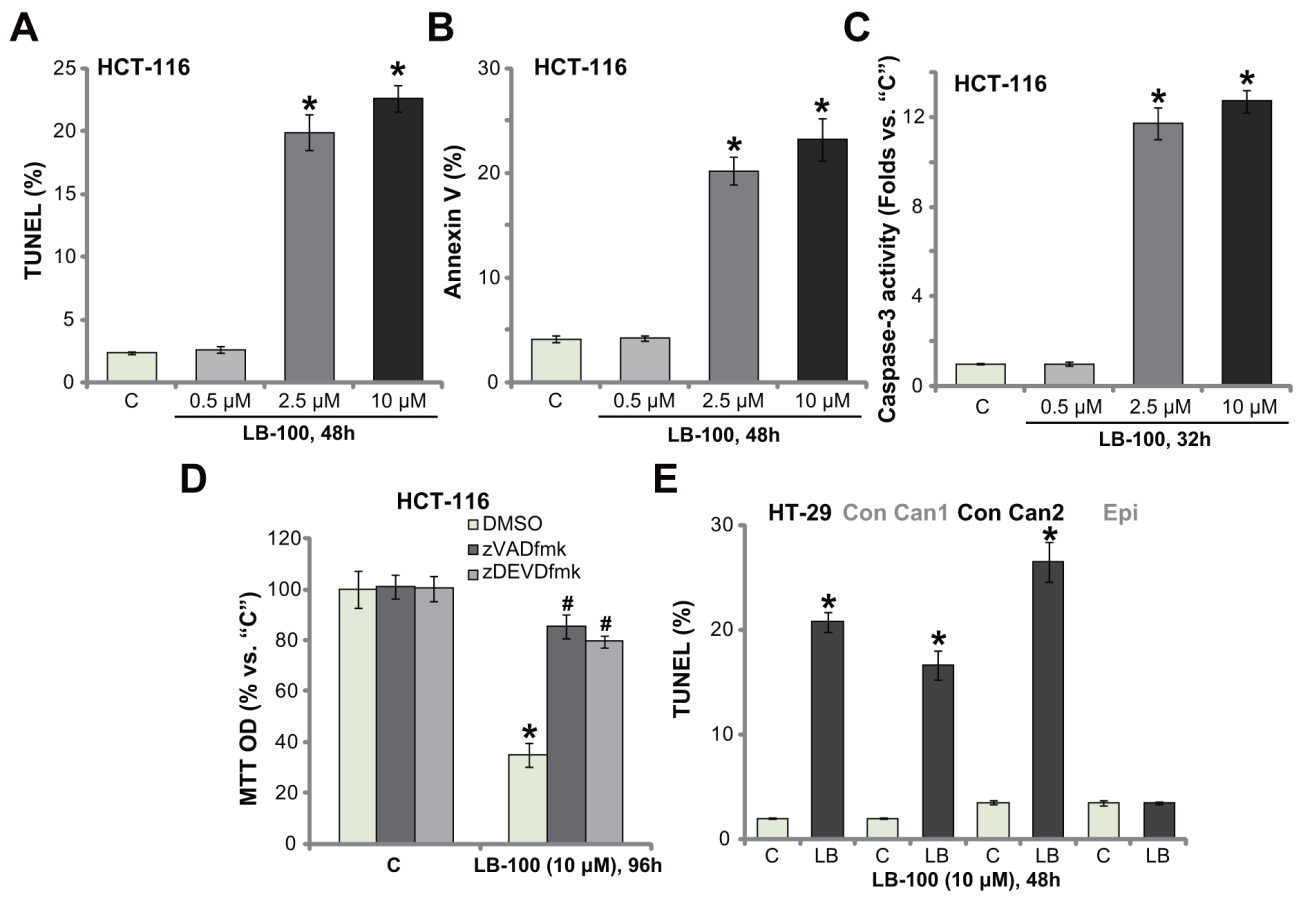
LB-100 activates apoptosis in CRC cells The established human CRC cells (HCT-116 and HT-29 lines), the primary-cultured human colon cancer cells (two lines, “Con Can1/2”), or the primary-cultured colon epithelial cells (“Epi”) were treated with LB-100 at designated concentration, cells were further cultured for applied time; Cell apoptosis were tested by the assays mentioned in the text **(A-C** and **E)**. For panel **(D)**, HCT-116 cells were co-treated with 50 μM of the caspase-3 inhibitor zDEVDfmk or the pan caspase inhibitor zVADfmk, and cell viability was tested by MTT assay. Error bars indicate standard deviation (SD). “C” stands for untreated control group. ^*^p<0.05 vs. “C” group. ^#^ *p*<0.05 vs. DMSO (0.1%) group **(D)**. Experiments in this figure were repeated three times, and similar results were obtained.

We next studied the potential activity of LB-100 on cell apoptosis. Various apoptosis assays were performed, including the DNA end labeling (TUNEL) assay, Annexin V FACS assay and the caspase-3 activity assay. As displayed, treatment with LB-100, at 2.5 μM and 10 μM, in HCT-116 cells significantly increased the percentage of TUNEL-stained cells (Figure 2A), Annexin V ratio (Figure 2B) and the caspase-3 activity (Figure 2C). These results indicated profound apoptosis activation in LB-100-treated cells (Figure 2A-2C). LB-100-induced apoptosis was again concentration-dependent (Figure 2A-2C), and it was yet in-effective when applied at a lower concentration (0. 5 μM, Figure 2A-2C). In order to block cell apoptosis, the caspase-based inhibitors were employed. As displayed, co-treatment with the caspase-3 inhibitor zDEVDfmk or the pan caspase inhibitor zVADfmk largely attenuated LB-100-induced viability (MTT OD) reduction in HCT-116 cells (Figure 2D). The two caspase inhibitors almost blocked LB-100-induced apoptosis activation (Data not shown). These results suggest that LB-100 activates caspase-dependent apoptosis to kill the CRC cells. It should be noted that significant apoptosis activation (TUNEL assay) was also observed in LB-100 (10 μM)-treated HT-29 cells and the primary human colon cancer cells (Figure 2E). TUNEL ratio was yet unchanged in the colon epithelial cells with same LB-100 treatment, further suggesting its selective response in cancerous cells (Figure 2E). These results demonstrate that LB-100 activates apoptosis in CRC cells.

### LB-100 induces G1-S arrest in CRC cells

**Figure 3:**
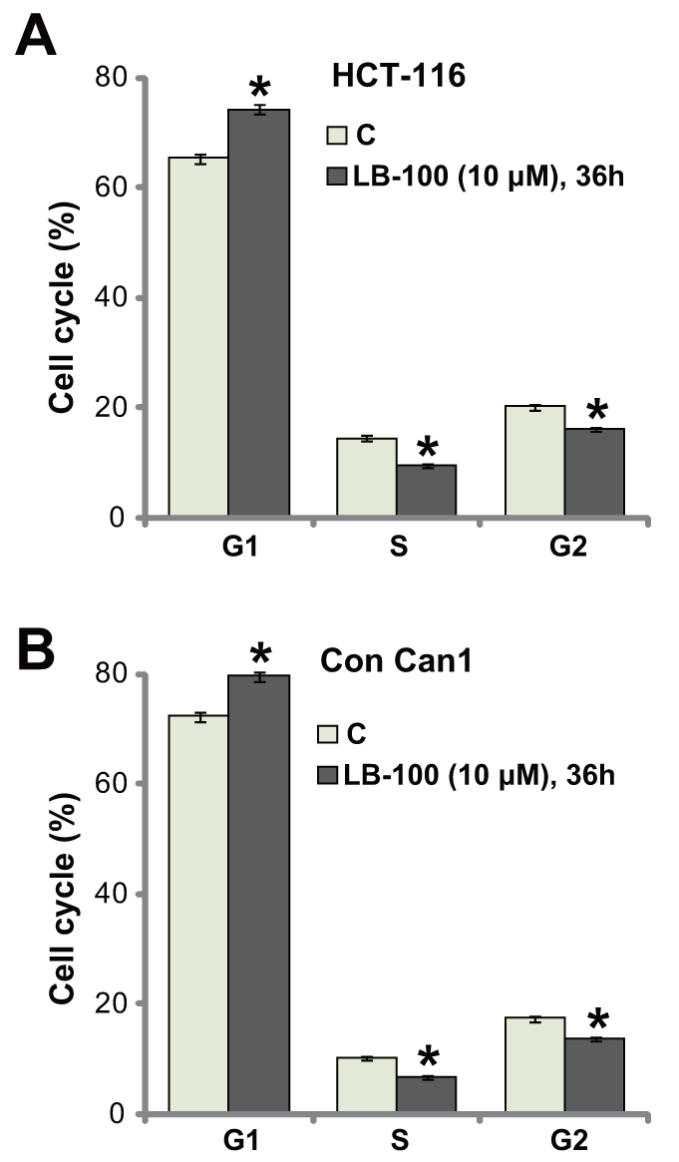
LB-100 induces G1-S arrest in CRC cells HCT-116 cells **(A)** or the primary-cultured human colon cancer cells (“Con Can1”, **(B)** were treated with LB-100 at 10 μM, cells were further cultured for 36 hours; Cell cycle distribution was tested by the FACS assay, percentages of G1-, S- and G2-phase cells were quantified (A and B). Error bars indicate standard deviation (SD). “C” stands for untreated control group. ^*^p<0.05 vs. “C” group. Experiments in this figure were repeated five times, and similar results were obtained.

The activity of LB-100 on cell cycle progression was also tested. HCT-116 cells were treated with LB-100 (10 μM), cells were further cultured for additional 36 hours, and FACS assay was employed to test cell cycle distribution. The quantified results in Figure 3A demonstrated that, following the LB-100 treatment, the percentage of G1-phase cells was increased, but the percentages S-phase and G2-phase cells were both decreased. These results indicate G1-S arrest by LB-100 treatment in HCT-116 cells, which should favor proliferation-inhibition and apoptosis. The very similar G1-S arrest result was also obtained in the LB-100-treated primary human colon cancer cells (Figure 3B). Thus, LB-100 induces G1-S arrest in CRC cells.

### LB-100 inhibits PP2A activity and activates AMPK signaling in CRC cells

**Figure 4:**
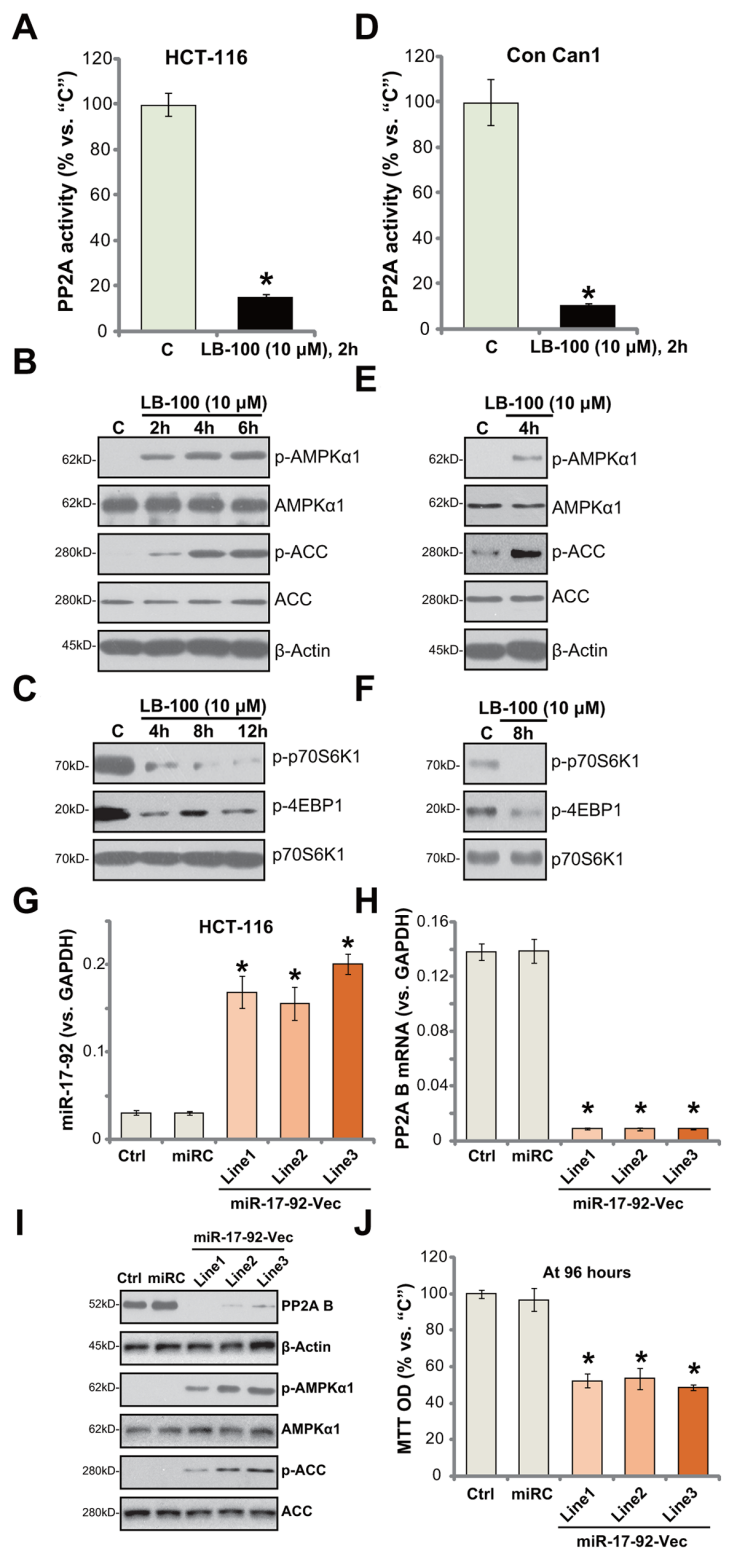
LB-100/miR-17-92 inhibits PP2A and activates AMPK signaling in CRC cells HCT-116 cells **(A-C)** or the primary-cultured human colon cancer cells (“Con Can1”, **D-F)** were treated with LB-100 at 10 μM, cells were further cultured for the designated time; Relative PP2A activity (A and D) was analyzed; Expressions of listed proteins were tested by Western blotting assay **(B, C, E** and **F)**. Stable HCT-116 cells, expressing miR-17-92 expression vector (“miR-17-92-Vec”, three lines, “Line1/2/3”), non-sense control microRNA (“miR”), or the parental control cells (“Ctrl”) were subjected to quantitative real-time PCR (“qRT-PCR”) assay of *miR-17-92* **(G)** and *PP2A B subunit* **(H)** as well as the Western blotting assay of listed proteins **(I)**; Cell survival at 96 hours was tested by MTT assay **(J)**. Error bars indicate standard deviation (SD). “C” stands for untreated control group. ^*^p<0.05 vs. “C” group (A and D). ^*^p<0.05 vs. “Ctrl” cells (G, H and J). Experiments in this figure were repeated three times, and similar results were obtained.

LB-100 is a novel PP2A inhibitor [13, 30–32]. Its effect on PP2A activity was examined. As displayed, treatment with LB-100 (10 μM) in HCT-116 cells largely inhibited PP2A activity (Figure 4A). Expression of PP2A was unchanged by the same LB-100 treatment (Data not shown). As discussed, recent studies have focused on the function of AMPK activation in suppressing human CRC cells [4, 21, 33–35]. Several anti-cancer agents were shown to activate AMPK signaling to inhibit CRC cells [4, 21, 33–35]. Forced-activation of AMPK, via adding AMPK activators or expressing genetic modified AMPKα, was able to efficiently inhibit CRC cells [4, 36]. Recent studies have proposed PP2A as a key AMPKα phosphotase [15, 16, 37].

Since LB-100 blocked PP2A activity (Figure 4A), we thus tested AMPK signaling in LB-100-treated cells. The Western blotting assay results in Figure 4B demonstrated that LB-100 (10 μM) induced profound AMPK activation in HCT-116 cells. AMPK activation was tested by phosphorylated (“p-“) AMPKα1 (at Thr-172) and its major downstream substrate protein, acetyl-CoA carboxylase (ACC, Ser-79) [19, 25]. One major consequence following AMPK activation is mTOR complex 1 (mTORC1) inhibition (See discussion below [19, 25]). Here, we demonstrate that treatment with LB-100 (10 μM) in HCT-116 cells largely inhibited phosphorylations of two primary mTORC1 substrates, including p70S6K1 and 4E-binding protein 1 (4E-BP1) [38–40], suggesting mTORC1 in-activation [41] (Figure 4C). Above-mentioned signalings were also tested in the primary human colon cancer cells. As demonstrated, LB-100 similarly blocked PP2A activation (Figure 4D), activated AMPK signaling (Figure 4E) and inhibited mTORC1 (Figure 4F) in the primary cancer cells. These results indicate that LB-100 blocks PP2A and activates AMPK signaling in HCT-116 cells.

### miR17-92-mediated silence of PP2A activates AMPK and inhibits HCT-116 cell survival

miRNAs (miRs) are capable of suppressing targeted-gene expression via binding to 3’-untranslated region (UTR) of targeted-mRNAs [42–44]. Previous studies have identified an anti-PP2A miR, miR-17-92 [45]. Here, a miR-17-92-expression vector was constructed and transfected to the HCT-116 cells. Three lines with the construct were established (“Line1/Line2/Line3”). As demonstrated, forced-expression of miR-17-92-vector indeed significantly increased miR-17-92 level in all three lines (Figure 4G). Consequently, PP2A (B subunit) mRNA (Figure 4H) and protein (Figure 4I) level was sharply reduced. AMPK activation, tested by p-AMPKα1/p-ACC, was increased (Figure 4I). Additionally, HCT-116 cell survival, tested by MTT assay, was inhibited in miR-17-92-expressing HCT-116 cells (Figure 4J). Thus, in line with the LB-200's data, miR17-92-mediated silence of PP2A also activated AMPK and inhibited HCT-116 cell survival. Notably, the nonsense microRNA control (“miRC”) failed to affect miR-17-92/PP2A expression (Figure 4G-4I) nor HCT-1116 cell survival (Figure 4J).

### AMPK activation is required for LB-100-induced cytotoxicity in HCT-116 cells

**Figure 5:**
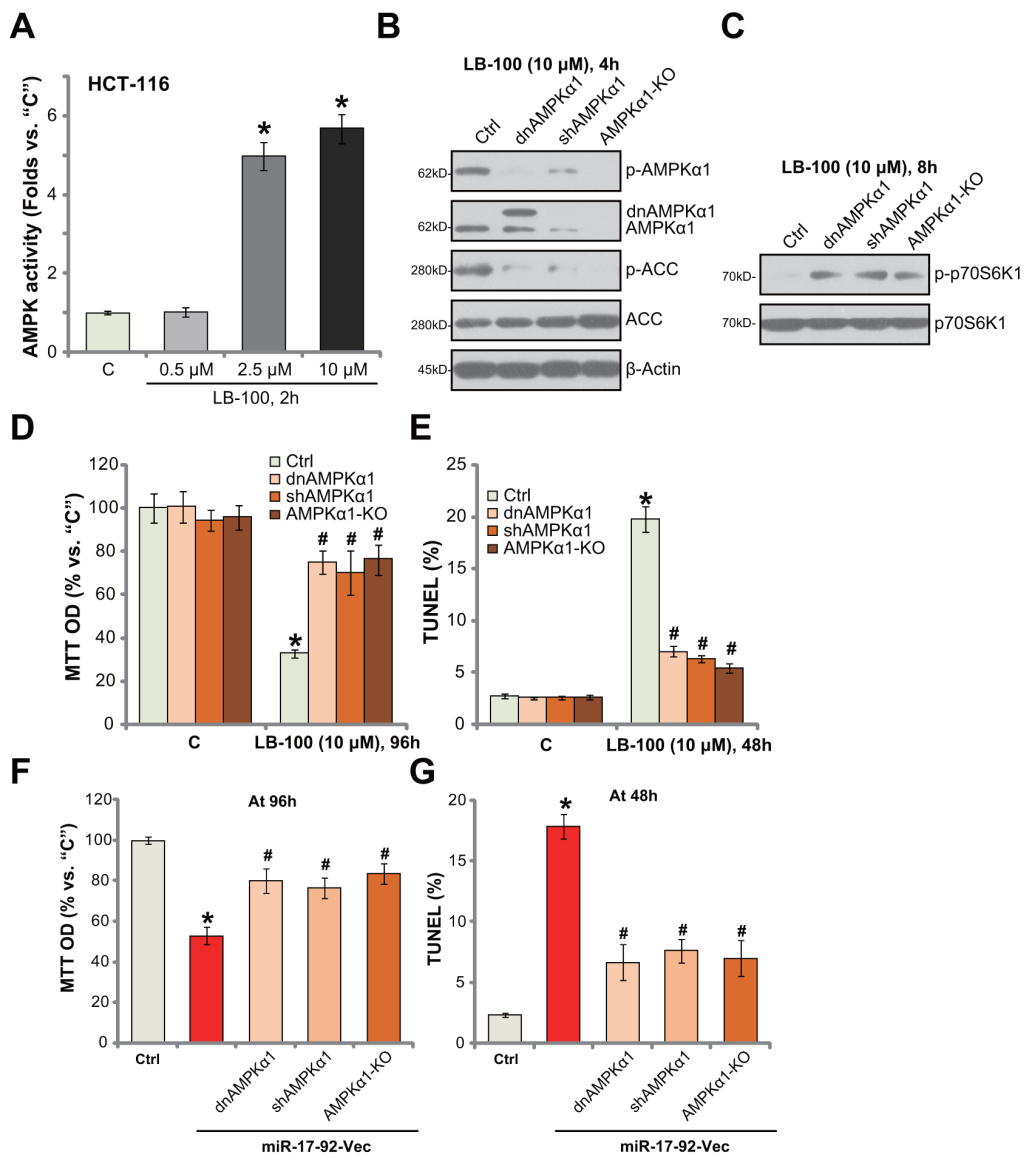
AMPK activation is required for LB-100/miR-17-92-induced cytotoxicity in HCT-116 cells HCT-116 cells were treated with LB-100 (at applied concentration), cells were further cultured for designated time; Relative AMPK activity was shown **(A)** Stable HCT-116 cells, expressing AMPKα1 shRNA (“shAMPKα1”), dominant negative AMPKα1 (T172A, Flag-tagged, “dnAMPKα1”) or CRISPR/Cas9 AMPKα1 (“AMPKα1-KO”), as well as the control parental HCT-116 cells (“Ctrl”) were treated with LB-100 (10 μM) or transfected with miR-17-92 expression vector (“miR-17-92-Vec”), cells were further cultured for designated time; Expressions of listed proteins were tested by Western blotting assay **(B** and **C)** Cell survival (MTT assay, **D** and **F**) and apoptosis (TUNEL assay, **E** and **G**) were also tested. Error bars indicate standard deviation (SD). “C” stands for untreated control group. ^*^p<0.05 vs. “C” group. ^#^ *p*<0.05 vs. “Ctrl” cells **(D** and **E)**. ^#^ *p*<0.05 vs. miR-17-92-Vec only cells **(F** and **G)**. Experiments in this figure were repeated three times, and similar results were obtained.

In order to further confirm that LB-100 activates AMPK signaling in CRC cells. AMPK activity was tested. As shown in Figure 5A, treatment with LB-100 (10 μM) in HCT-116 cells significantly increased AMPK activity in HCT-116 cells. The effect by LB-100 was again dose-dependent (Figure 5A). Next, genetic strategies were applied to block AMPK activation. First, a dominant negative AMPKα1 (T172A, from Dr. Wu [4]) was introduced to HCT-116 cells, which in-activated AMPKα1 (Figure 5B). Secondly, the lentiviral AMPKα1 shRNA was added to HCT-116 cells to stably knockdown AMPKα1 (Figure 5B). Third, the CRISPR/Cas9 method was utilized to knockout AMPKα1 [46] in HCT-116 cells (Figure 5B). As displayed, AMPKα1 mutation (T172A), knockdown (by targeted-shRNA) or complete knockout (by CRISPR/Cas9) almost completely blocked LB-100-induced AMPK activation, or AMPKα1/ACC phosphorylations (Figure 5B). Consequently, mTORC1 activation, tested by p-p70S6K1, was restored (Figure 5C). Remarkably, AMPKα1 mutation, shRNA knockdown or complete knockout largely attenuated LB-100 (10 μM)-induced HCT-116 cell viability (“MTT OD”) reduction (Figure 5D) and apoptosis activation (TUNEL staining, Figure 5E). These results show that genetic in-activation of AMPK largely attenuated LB-200-induced cytotoxicity against HCT-116 cells. Thus, AMPK activation is required for LB-100-induced cytotoxicity in HCT-116 cells.

### AMPK activation is required for miR17-92-induced cytotoxicity against HCT-116 cells

Further studies showed that forced-expression of miR17-92-induced HCT-116 cell death (MTT OD reduction, Figure 5F) and apoptosis (TUNEL increase, Figure 5G) were also largely attenuated with AMPKα mutation (T172A), silence or knockout. These results suggest that AMPK activation is also required for miR17-92-induced cytotoxicity against HCT-116 cells.

### LB-100 administration activates AMPK signaling and inhibits HCT-116 tumor growth in nude mice

**Figure 6:**
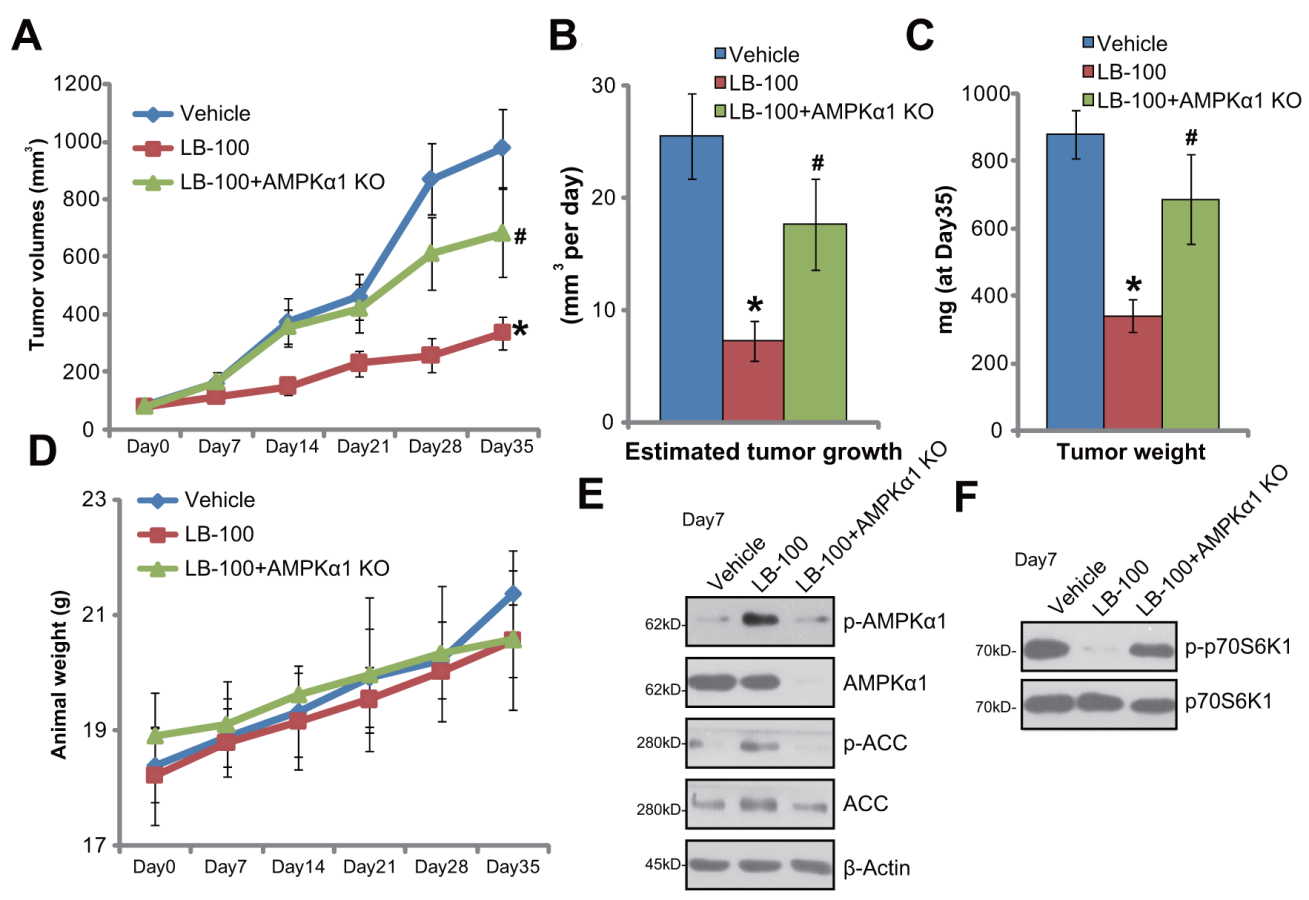
LB-100 administration activates AMPK signaling and inhibits HCT-116 tumor growth in nude mice Weekly tumor growth curve of xenografts (from AMPKα1 knockout or control HCT-116 cells) **(A)** and mice body weight curve **(D)** with indicated treatment: Saline (“Vehicle”, *i.p.*), or LB-100 (5 mg/kg, *i.p.* daily, for 21 days), were shown; Estimated daily tumor growth **(B)** and tumor weights (at Day-35, **C**) were also shown; Seven days after initial LB-100 treatment, one tumor of each group was removed; Tumor tissues were subjected to Western blotting assay of listed proteins **(E** and **F)**. Error bars indicate standard deviation (SD). ^*^ *p* < 0.05 vs. “Vehicle” group. ^#^ *p* < 0.05 vs. control tumors.

The LB-100's activity on CRC growth *in vivo* was tested. HCT-116 cells, with or without AMPKα1, were injected *s.c.* to the nude mice. HCT-116 xenografts were then established. Weekly tumor growth curve results in Figure 6A displayed that daily intraperitoneally (*i.p.*) administration of LB-100 (5 mg/kg body weight) potently inhibited growth of HCT-116 tumors in the mice. The volume of LB-100-treated tumors was markedly lower than that of the vehicle control mice (Figure 6A). The estimated daily tumor growth (in mm^3^ per day), which was calculated by (tumor volume at day-35—tumor volume at day-0)/35, was also significantly decreased by LB-100 administration (Figure 6B). Additionally, the average tumor weight at day-35 was dramatically lower in the LB-100-treated group (Figure 6C). These results indicated that LB-100 *i.p.* administration inhibited HCT-116 tumor growth in nude mice. The mice body weights, which reflected animals’ general health condition, were not significantly changed by LB-100 administration (Figure 6D). No significant or apparent toxicities were observed in the experimental mice.

Notably, LB-100-induced anti-tumor activity *in vivo* was largely compromised against tumors that were derived from AMPKα1-knockout (by CRISPR/Cas9 method) HCT-116 cells (“+AMPKα1 KO”, Figure 6A-6C). These results suggest that AMPK activation should also be required or LB-100-induced activity *in vivo*. Indeed, when analyzing signaling changes in tumor tissues, we demonstrate that LB-100 administration activated AMPK (AMPKα1/ACC phosphorylations) in HCT-116 tumors (Figure 6D), which was followed by mTORC1 (p-p70S6K1) inhibition (Figure 6F). Tumors-derived from AMPKα1-knockout cells showed depleted AMPKα1 (Figure 6E). LB-100-induced AMPK activation (Figure 6E) and mTORC1 inhibition (Figure 6F) were almost abolished in AMPKα1-knockout tumors. Together, we conclude that LB-100 administration activates AMPK and inhibits HCT-116 tumor growth in nude mice.

## DISCUSSION

AMPKα1 phosphorylation at Thr-172 is vital for AMPK activation [20, 47, 48]. Studies have been focusing on the upstream mechanisms of phosphorylation of AMPKα1 [26]. A number of potential AMPKα1 kinases have been identified. These kinases, including LKB1 (Liver kinase B1) [48] and CaMKK (calcium/calmodulin-dependent protein kinase kinase) [49], directly phosphorylate AMPKα1 at Thr-172, leading to profound AMPK activation. On the other hand, the mechanisms of AMPKα1 de-phosphorylation are largely unknown. Several potential AMPKα1 phosphatase were proposed [50]. One key AMPKα1 phosphatase is PP2A [15, 37]. In the current study, we show that targeted inhibition of PP2A by LB-100 activated AMPK signaling in CRC cells. Notably, AMPKα in-activation, silence or complete depletion via the described methods didn't result in total abolition of LB-100-induced cytotoxicity against HCT-116 cells (See Figure 5). These results suggest that other signalings, besides AMPK activation, could also be responsible for LB-100's actions in CRC cells. These results are not surprising, as PP2A could de-phosphorylate substrate kinases other than AMPK [10, 51]. It should also be noted that miR-17-92-mediated silence of PP2A also activated AMPK and induced HCT-116 cell death. Such effects were again largely attenuated by AMPKα mutation, silence or complete knockout.

Dysregulation of mTOR signaling has been detected in CRC tissues [52, 53]. Sustained and over-activation of mTOR in CRC is positively involved in a number of key oncogenic behaviors, including uncontrolled cancer cell survival, proliferation and migration, as well as chemo-resistance and angiogenesis [52, 53]. Thus, mTOR over-activation represents an important oncotarget of CRC treatment [3, 4]. AMPK activation shall lead to mTORC1 in-activation, thus inhibiting CRC and other cancer cells. Tuberous sclerosis 2 (TSC2) phosphorylation and activation by activated AMPK would lead to downstream mTORC1 in-activation [54–56]. Meanwhile, AMPK is also shown to directly phosphorylate and in-activate Raptor, a key mTORC1 component, to shut down mTORC1 activation [57, 58].

In the current study, we show that AMPK activation by LB-100 also inhibited mTORC1 activation in CRC cells. Phosphorylations of mTORC1 substrates, including p70S6K1 and 4E-BP1, were dramatically inhibited in LB-100-treated cells. Notably, LB-100-induced mTORC1 inhibition was almost completely reversed with AMPKα1 mutation, shRNA knockdown or complete knockout. Thus, we propose that blockage of PP2A by LB-100 activates AMPK to inhibit mTORC1 activation in CRC cells. This mechanism could a primary reason of CRC cell inhibition. These results might also explain the in-effectiveness of LB-100 against human colon epithelial cells, as the normal cells displayed extremely low level of mTORC1 activation [59].

## MATERIALS AND METHODS

### Reagents

LB-100 was provided by Selleck (Beijing, China). The caspase-3 inhibitor zDEVDfmk and the pan caspase inhibitor zVADfmk were purchased from Sigma (Nanjing, China). All the antibodies of this study were purchased from Cell Signaling Technology (Beverly, MA). The tissue-culture reagents were provide by Gibco (Nanjing, China).

### Culture of established cell lines

The established human CRC cell lines, HCT-116 and HT-29, were purchased from the iBS Cell Bank of Fudan University (Shanghai, China). Cells were cultured in DMEM medium, plus 6% FBS. DNA fingerprinting and profiling were performed to distinguish the cell line from possible cross-contamination.

### Primary human colon cancer cells and colon epithelial cells

The protocols of isolation and culture of human colon cancer cells as well as the primary colon epithelial cells were described in detail previously [60, 61]. In brief, the colon cancer issues and the surrounding normal colon epithelial tissues from patients (two male, 49 and 59 years old) were obtained, washed, and digested. The single cell suspensions were pelleted, and resuspended in the described medium plus growth factors for the primary human cells [61]. A total of two primary human colon cancer cell lines and one colon epithelial cell line were established. Written-informed consent was obtained from each patient. The protocols were approved by the IRB (Institutional Review Board) and Ethics Committee of all authors’ institutions, and experiments were conducted according to the principles of Declaration of Helsinki.

### Cell viability assay

The cell survival was always tested by the 3-[4,5-dimethylthylthiazol-2-yl]-2,5 diphenyltetrazolium bromide (MTT) (Sigma, Nanjing, China) assay according to the attached protocol [61]. The MTT OD of treatment cells was always expressed as the percentage of that of untreated control cells.

### Colony formation assay

HCT-116 cells were initially plated at 5 × 10^3^ cells per 10-cm tissue culture dish. LB-100-containing medium was renewed every two days. After 10 days of incubation, the remaining HCT-116 colonies were stained and manually counted.

### Cell proliferation assay

The simple cell counting assay and BrdU incorporation ELISA assay were performed to test cell proliferation. The detailed protocols were described previously [4, 62].

### Annexin V FACS analysis

The Annexin V In Situ Cell Apoptosis Detection Kit (Roche, Indianapolis, IN) [61, 63] was employed to test cell apoptosis. In brief, following the applied LB-100 treatment, cells were washed, fixed and stained with Annexin V (2.5 μg/mL) and propidium iodide (PI) (2.5 μg/mL). The cell apoptosis ratio was reflected by Annexin V^+/+^/PI^-/-^ plus Annexin V^+/+^/PI^+/+^ percentage detected by fluorescence-activated cell sorting (FACS) counter (Beckman Coulter, Shanghai, China).

### Apoptosis assays

Other apoptosis assays, including TUNEL assay and caspase-3 activity, were performed as previously described [4].

### Cell cycle analysis

Following the applied LB-100 treatment, CRC cells were labeled with 10 μM EdU (Biyuntian, Nanjing, China) for 1 hour and were then fixed. Cells were then analyzed by flow cytometry (Beckman Coulter, Shanghai, China). Cell cycle distribution was analyzed with Kaluza software (Beckman Coulter).

### Western blotting assay

Cells and tumor tissues were lysed by the commercial available RIPA lysis buffer (Biyuntian) plus Complete Protease Inhibitor Cocktail (Roche) and phosphatase inhibitors (Roche). Quantified lysate proteins (40 μg of each condition) were separated by the 7.5-10 % SDS-PAGE gels (Biyuntian), and were transferred onto the polyvinylidene fluoride (PVDF) membrane. The blot was then blocked, and were probed overnight with designated primary antibody. The corresponding horseradish peroxidase (HRP)-conjugated secondary antibody was then added. Super-Signal West Pico Chemiluminescent Substrates (Thermo Scientific, Shanghai, China) were added to visualize the targeted protein band (based on the molecular weight), under the X-ray film development.

### PP2A phosphatase activity assay

After the designated LB-100 treatment, CRC cells were washed and lysed in the described RIPA lysis buffer. The supernatants of 50 μg of total cellular proteins were assayed with the PP2A Phosphatase Assay Kit (Millipore) by the attached protocol. The PP2A activity data were presented as percentage of relative PP2A activity compared with control.

### AMPK activity assay

Following the LB-100 treatment, cell lysates were achieved. AMPK was firstly immunoprecipitated with anti-pan-AMPKα antibody. The AMPK activity was determined in kinase assay buffer (see previous study [64]) plus AMP-[γ-^32^P] ATP mixture, and SAMS peptide (HMRSAMSGLHLVKRR) [64]. The reaction was terminated by spotting the reaction mixture on phosphocellulose paper (P81), which was extensively washed with 150 mM of phosphoric acid. The radioactivity was measured with scintillation counter. AMPK activity in the treatment group was always normalized to that of the untreated control group.

### AMPKα1 shRNA

The lentiviral particles, with human AMPKα1 shRNA or scramble non-sense control shRNA, were obtained from Santa Cruz Biotech (Nanjing, China). The lentivirus was added to HCT-116 cells for 24 hours. Puromycin (3.0 μg/mL, Sigma) was then added, which helped to select the stable cells.

### AMPKα1 mutation

The dominant negative AMPKα1 (dn-AMPK-α1, T172A, Flag-tagged) construct was from Dr. Wu [4]. The transfection of mutant AMPKα1 by Lipofectamine 2000 was described previously [4]. Neomycin (1 μg/mL) was added to select the stable cells. The mutant AMPKα1 expression was confirmed by Western blotting assay.

### CRISPR/Cas9-mediated AMPKα1 knockout

The small guide RNA (sgRNA) targeting human AMPKα1 was through the Optimized Crispr Design application from Dr. Zhang's lab (http://crispr.mit.edu/), which was inserted into the lentiCRISPR plasmid (Addgene plasmid 49535, Shanghai, China). The plasmid was transfected to HCT-116 cells using the described protocol [46]. AMPKα1 expression in the stable cells was confirmed by Western blotting assay.

### Forced miR-17-92 expression

The pSuper-GPF-puro-miR-17-92 expression vector, encoding miR-17-92 (based on the sequence reported previously [65, 66]), was designed, synthesized and sequence-verified by Shanghai Genepharm Co. (Shanghai, China). The HCT-116 cells were transfected with miR-17-92 expression vector or the scramble miRNA control (“miR-C”) via the Lipofectamine 2000 reagent (Invitrogen, Nanjing, China). Forty-eight hours after transfection of miR, stable cells were selected by puromycin (0.5 μg/mL, Sigma).

### RNA isolation and RT-PCR

Total cellular RNA was extracted by the RNeasy Mini Kit (Qiagen, Wuxi, China). A total of 600 ng RNA per treatment was reverse-transcribed by commercial SYBR green kit (TOYOBO, Japan). Quantitative real-time PCR (“qRT-PCR”) was performed through the ABI-7500 fast PCR system (Applied Biosystems, Shanghai, China) [67, 68]. PP2A regulatory (B subunit) mRNA primers were reported early [45, 69]. For miR analysis, miR was converted to cDNA from 600 ng of total RNA using the First-Strand Synthesis Kit (SABiosciences, Frederick, MD). miR-17-92 analysis was performed through qRT-PCR assay using the miR-17-92 primers (SABiosciences) [65, 66]. miR-17-92 level was also normalized to GAPDH.

### Xenograft tumor assay

BALB/c nude mice (17.8-18.5 grams, 4-5 week old, all female) were injected subcutaneously (*s.c.*) in the right flanks with 1 × 10^6^ HCT-116 cells (per mouse). Animals were housed in temperature- and humidity-controlled cages, with free access to water and rodent food on a 12-h light/dark cycle. After a tumor volume of 80 mm^3^ was reached, LB-100 were injected intraperitoneally (*i.p.*) daily for a total of 21 days. Control mice were injected with equal quantity of vehicle. Tumor volumes were recorded weekly, calculated via the following formula: π/6 × larger diameter × (smaller diameter)^2^. All animal studies were performed in accordance with the standards of ERB and IACUC of all authors’ institutions.

### Statistical analysis

The results were expressed as the mean ± standard deviation (SD). Ordinary one-way ANOVA test was employed for comparison between groups. *p* < 0.05 was considered as statistically significant.

## CONCLUSION

The previous cancer studies have suggested that PP2A inhibition is likely to be most effective for cancer therapy when combined with traditional cytotoxic agents [14, 31, 32]. The results of this study show that PP2A inhibition by LB-100 or miR-17-92 may have significant anti-CRC cell activity *in vitro* and *in vivo*. LB-100 or miR-17-92 could be further tested as promising anti-CRC agents.
